# Integrative analysis of the epigenetic basis of muscle-invasive urothelial carcinoma

**DOI:** 10.1186/s13148-018-0451-x

**Published:** 2018-02-12

**Authors:** Thomas Sanford, Maxwell V. Meng, Reema Railkar, Piyush K. Agarwal, Sima P. Porten

**Affiliations:** 1 Department of Urology, University of California, Mail code 1695, 550 16th Street, 6th Floor, San Francisco, CA 94143 USA; 2 Urologic Oncology Branch, Center for Cancer Research, National Cancer Institute, Building 10—Hatfield CRC, Room 2-5952, Bethesda, MD 20892-1210 USA

**Keywords:** Epigenetics, Urothelial carcinoma, Integrative analyses

## Abstract

**Background:**

Elucidation of epigenetic alterations in bladder cancer will lead to further understanding of the biology of the disease and hopefully improved therapies. Our aim was to perform an integrative epigenetic analysis of invasive urothelial carcinoma of the bladder to identify the epigenetic abnormalities involved in the development and progression of this cancer.

**Methods:**

Pre-processed methylation data and RNA-seq data were downloaded from The Cancer Genome Atlas (TCGA) and processed using the R package TCGA-Assembler. An R package MethylMix was used to perform an analysis incorporating both methylation and gene expression data on all samples, as well as a subset analysis comparing patients surviving less than 2 years and patients surviving more than 2 years. Genes associated with poor prognosis were individually queried. Pathway analysis was performed on statistically significant genes identified by MethylMix criteria using ConsensusPathDB. Validation was performed using flow cytometry on bladder cancer cell lines.

**Results:**

A total of 408 patients met all inclusion criteria. There were a total of 240 genes differentially methylated by MethylMix criteria. Review of individual genes specific to poor-prognosis patients revealed the majority to be candidate tumor suppressors in other cancer types. Pathway analysis showed increase in methylation of genes involved in antioxidant pathways including glutathione and NRF2. Genes involved in estrogen metabolism were also hypermethylated while genes involved in the EGFR pathway were found to be hypomethylated. EGFR expression was confirmed to be elevated in six bladder cancer cell lines.

**Conclusions:**

In patients with invasive urothelial carcinoma, we found differential methylation in patients with better and worse prognosis after cystectomy. Differentially methylated genes are involved in many relevant oncologic pathways, including EGFR and antioxidant pathways, that may be a target for therapy or chemoprevention.

**Electronic supplementary material:**

The online version of this article (10.1186/s13148-018-0451-x) contains supplementary material, which is available to authorized users.

## Background

Urothelial carcinoma of the bladder is the eighth most common form of cancer in the USA and the fourth most common type of cancer in men [1]. Although the majority of urothelial carcinoma of the bladder is diagnosed at a non-invasive stage, 30–40% of cases will progress to invade the deeper muscle layer of the bladder [2], at which point the long-term disease-specific survival is 50–70% [3, 4].

Multiple studies have shown that DNA methylation, the process by which a methyl group is added to a cytosine residue of a cytosine-phosphate-guanine (CpG) dinucleotide, plays an important role in the initiation and progression of bladder cancer [5–7]. DNA methylation represents a promising target for therapy since DNA methylation is a reversible process that does not alter the content of DNA [8].

High-throughput methods have been employed in bladder cancer to evaluate the epigenetic alterations involved in the development and progression of this disease [5, 9]. However, many methylation events found to be statistically significant using high-throughput screening methods are not correlated with gene expression changes [10]. There is a need for high-throughput approaches that integrate data across multiple platforms to determine the epigenetic events that are most likely to be involved in bladder cancer [11].

The Cancer Genome Atlas (TCGA) project demonstrated impressive diversity in both genetic and epigenetic alterations within patients who have muscle-invasive urothelial carcinoma of the bladder [12]. The multiple platforms utilized within TCGA make it possible to perform analyses integrating data from multiple sources to identify specific abnormalities most likely to contribute to oncogenic processes. In this study, we utilized an integrative approach to evaluate the epigenetic processes that may be most important in development and progression of invasive urothelial carcinoma.

## Methods

### Data acquisition and preprocessing

All data were obtained from bladder cystectomy specimens from The Cancer Genome Atlas Project [13]. Specimen acquisition and processing is described in detail in the original TCGA publication, but briefly: specimens were placed in optimum cutting temperature media and frozen. Normal tissues (*N* = 21) were taken from the cystectomy specimens 2 cm away from tumor. All tumors included in TCGA had less than 50% of variant (non-urothelial) histology.

Methylation was evaluated on the Illumina Infinium HumanMethylation450 platform, which assesses 482,421 CpG sites throughout the genome [14]. Level 3 methylation data were downloaded from the TCGA data portal using the TCGA-Assembler *DownloadMethylationData* function [15]. Level 3 data consist of pre-processed data via TCGA pipelines in the form of beta values, which are a ratio between methylated probe intensities and total probe intensities (https://cancergenome.nih.gov/abouttcga/aboutdata/datalevelstypes). Probe-level data was condensed to a summary beta value for each gene using the *Methylation450_single_value* function in TCGA-Assembler, which calculates the average methylation value for all CpG sites associated with a gene. Level 3 RNA-seq data were also obtained from the TCGA data portal using the TCGA-Assembler function *DownloadRNASeqData*. The functions DGEList and calcNormFactors functions from the edgeR package were used to normalize the data [16]. The *voom* function from the limma package was then used to transform the RNA-seq data for linear modeling [17]. Both tumor tissue and normal tissue were processed in an identical manner.

### Clinical data

Clinical data for TCGA patients were obtained via the TCGA data portal. To augment and validate the pathologic data (of which not all variables were complete), pathology reports were downloaded from cbioportal.com for all patients in the TCGA provisional dataset and pathologic data were individually reviewed. All patients included in TCGA had muscle-invasive disease (pT2-T4). Of the 412 patients in the dataset, 408 had complete clinical, gene expression, and methylation data. A total of 101 patients met inclusion criteria for survival of at least 2 years after cystectomy. A total of 142 patients died within 2 years after cystectomy. This left a total of 165 patients with inadequate follow-up to be included in the survival analysis. Clinical data were partitioned into three separate groups for analysis. First, we performed analysis of the entire dataset (*N* = 408). Next, we performed analysis of patients who survived more than 2 years after cystectomy versus patients who died within 2 years of cystectomy.

### Integrative analysis

The R package MethylMix was used to perform an analysis integrating methylation data and gene expression (RNA-seq) data [18]. MethylMix is a program designed to identify methylation events that are correlated with gene expression [19]. There are three parts to the MethylMix analysis: first, methylation data are correlated with gene expression data to identify methylation events that results in gene expression changes—only genes passing the correlation filter are selected for further analysis; second, a beta mixture model is used to define a methylation state across a large number of patients, precluding the need for arbitrary thresholds; and third, a Wilcoxon rank sum test is used to compare DNA methylation states in tumor samples versus normal samples [18]. Multiple testing is accounted for using a *q* value cutoff of 0.05. The end result is a differential methylation (DM) value where a positive DM value signifies hypermethylation and a negative DM value signifies hypomethylation.

### Pathway analysis

Pathway analysis was performed with ConsensusPathDB [20]. ConsensusPathDB utilizes a hypergeometric test to evaluate for over-represented pathways based on the imputed gene list. The over-representation analysis function was utilized imputing the HUGO Gene Nomenclature Committee (HGNC) unique identifiers for each gene list. The following pathway databases were selected for our analysis: Inoh, Pid, Biocarta, Netpath, Humancyc, Kegg, Wikipathways, Smpdb, Pharmgkb, Ehmn, and Signalink. We used the default settings: minimum overlap and *p* value cutoff of 0.01.

Pathway analysis was performed using the gene lists found to be statistically significant by MethylMix. Lists of hypermethylated genes were analyzed separately from lists of hypomethylated genes for the followed groups: all patients, patients who survived at least 2 years after cystectomy, and patients who died within 2 years of cystectomy.

### Experimental validation in bladder cancer cell lines

The following bladder cancer cell lines were obtained from ATCC (Manassas, VA, USA): ScaBER (HTB-3), HT1376 (CRL-1472), SW780 (CRL-2169). Dr. David McConkey kindly gifted three additional cell lines UMUC-5, UMUC-1, and 253J (University of Texas, MD Anderson Cancer Center). All the cell lines were grown in the same media, which was minimum essential media (MEM) (Life Technologies) supplemented with 10% fetal bovine serum, 1% penicillin/streptomycin, and 1% GlutaMAX (Life Technologies). The presence of surface EGFR on the cell lines listed above was determined by flow cytometry. For each cell line, a single cell suspension was obtained after trypsinzation (~ 1 × 10^6^ cells/tube), which was incubated in the presence of phycoerythrin(PE)-tagged rat monoclonal antibody to human EGFR (Abcam) or PE-tagged rat IgG2a kappa monoclonal antibody (isotype control; Abcam). The cells were allowed to incubate for 30 min at 4 °C in the antibody solution. We washed the cells to remove all unbound antibody using phosphate-buffered solution. Fluorescence was measured on a FACS Calibur flow cytometer (BD BioSciences).

### Statistical analysis

Continuous variables were evaluated using the Student *t* test. Categorical variables were evaluated using chi-squared. *p* value less than 0.05 was considered statistically significant. All analyses were performed using R v 3.3.2. Flow cytometry data were analyzed using FlowJo (Treestar Inc.).

## Results

### Clinical data

Clinical characteristics are summarized in Table 1 and represent the three groups used for analysis. There was a statistically significant difference between the groups with respect to age, with patients surviving more than 2 years being younger. However, there were no significant differences between the groups for T stage [21].

**Table 1:** Clinical data

Characteristic	Total	Survive > 2 years after surgery	Death < 2 years of surgery	Inadequate follow-up	*p* value
Cohort size	408	101	142	165	
Mean age, years (SD)	68.0 (10.6)	66.2 (10.7)	70.6 (9.5)	69.3 (10.7)	< 0.01
Gender					
Women (%) Men (%)	106 (26%) 302 (74%)	24 (24%) 77 (76%)	41 (29%) 101 (71%)	51 (31%) 114 (69%)	0.45
T stage					
pT2 pT3 PT4 NA	120 (29%) 196 (48%) 59 (14%) 33 (8%)	37 (37%) 44 (44%) 11 (11%) 9 (9%)	24 (17%) 76 (54%) 29 (20%) 13 (9%)	43 (26%) 82 (50%) 25 (15%) 15 (9%)	0.06

*p* value determined by ANOVA for continuous variables and chi-square for categorical variables

### MethylMix analysis: hypermethylated versus hypomethylated genes by prognostic category

**Fig. 1:**
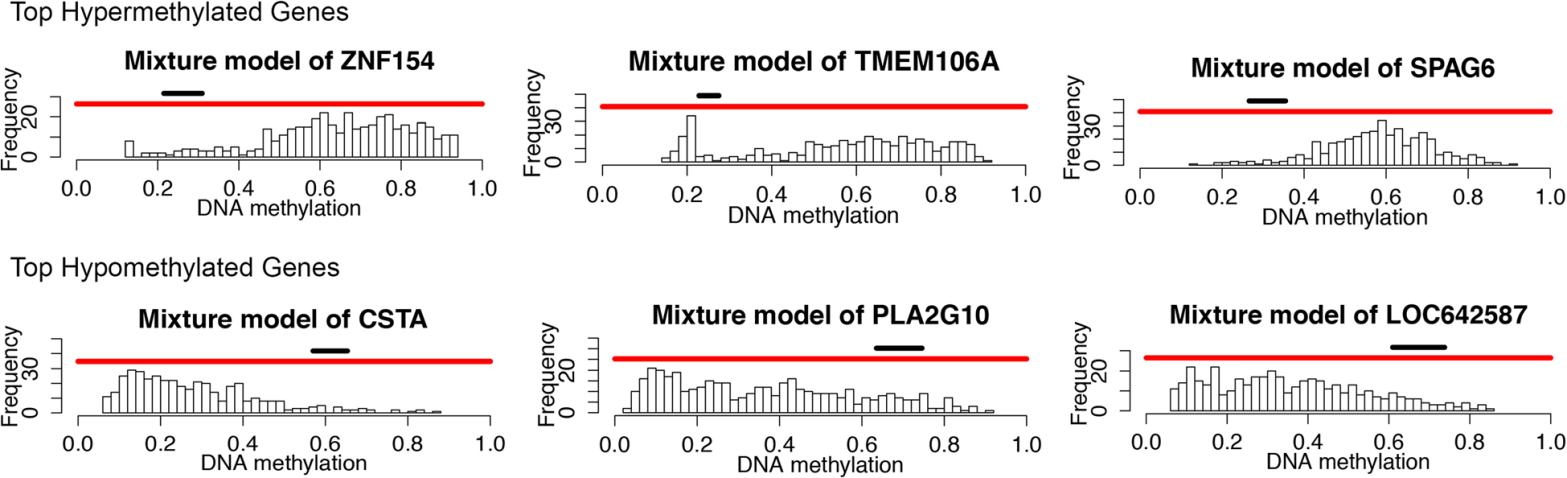
Summary of top hypermethylated and top hypomethylated genes. The red line demarcates the distribution of methylation in tumor samples and normal samples. The histogram (below red line) demonstrates the distribution of methylation in tumor samples (denoted as beta values where higher beta values represent greater methylation). The horizontal black bar above the red line represents the distribution of methylation values in the normal samples

**Fig. 2:**
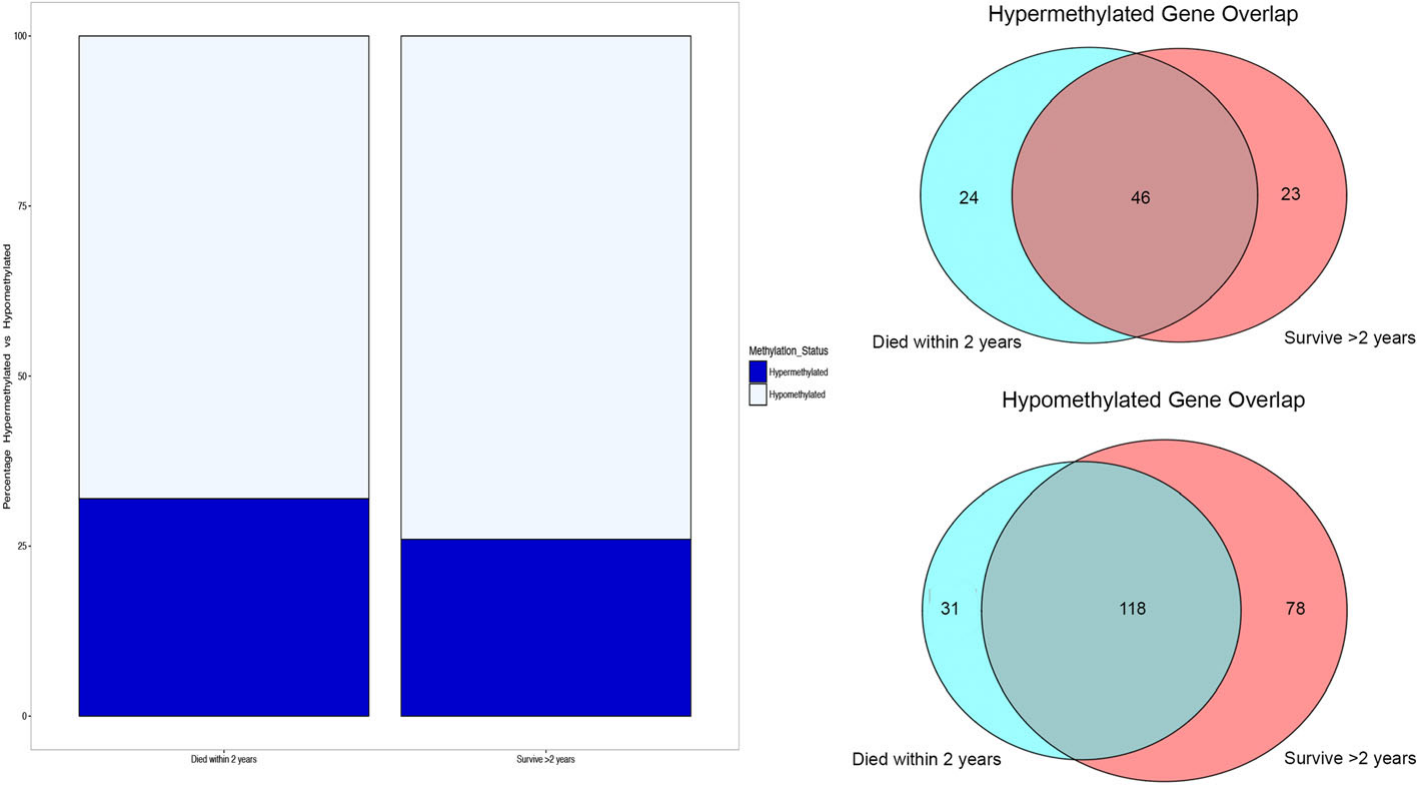
Of the 220 statistically significant genes for patients who died within 2 years, 32% were hypermethylated. Of the 266 statistically significant genes for patients who survived more than 2 years after cystectomy, 26% were hypermethylated and the remainder of the genes were hypomethylated. For both hypermethylated and hypomethylated genes, the majority of genes were shared by the better and worse prognosis groups. However, there were some genes with unique methylation status between the two groups, and there were more genes uniquely hypermethylated in the group that did not survive 2 years compared with the group that survived at least 2 years (*p* = 0.02). Identities of all genes significant by MethylMix are included in Additional file 1: Table S1

A total of 240 genes were differentially methylated when comparing tumor to normal by MethylMix criteria for all 408 patients. Example of differential methylation of tumor samples compared with normal samples is demonstrated in Fig. 1. Of these genes, 70 genes (29%) were hypermethylated and the remainder of the genes were hypomethylated. When MethylMix analysis was performed on the subset of patients surviving at least 2 years after cystectomy, a total of 266 genes reached statistical significance of which 69 (26%) were hypermethylated. There were a total of 220 significant genes when MethylMix was performed on the cohort of patients who died within 2 years of cystectomy, of which 70 (32%) were hypermethylated (Fig. 2). There was a slightly higher proportion of genes hypermethylated in patients in the worse prognosis group (survive < 2 years) when compared with the group with better prognosis (survive > 2 years), but this difference was not statistically significant (*p* = 0.18).

When the differentially methylated genes in the group that survived more than 2 years were compared with differentially methylated genes in the group that survived less than 2 years, nearly half of the genes (49%) overlapped between the better and worse prognosis cohorts. There were a total of 24 genes (26%) that were uniquely hypermethylated in the worse prognosis group and 23 genes (25%) uniquely hypermethylated in the better prognosis group. When the overlap of hypomethylated genes was evaluated, there was 52% overlap. The proportion of genes unique to the poor-prognosis group in the hypomethylated group was less than the hypermethylated group (13 versus 25%, respectively, *p* = 0.02). Identities of all genes significant by MethylMix are included in Additional file 1: Table S1.

### Evaluation of genes hypermethylated in patients surviving < 2 years

We sought to evaluate the genes hypermethylated in the patients unique to patients surviving less than 2 years to evaluate any potential biologic effect (Additional files 2, 3, and 4). We began by performing a literature search of PubMed for each gene using the terms “[GENE NAME]”, “methylation”, “cancer.” Of the 24 genes unique to patients who died within 2 years of surgery, 19 had been described as being candidate tumor suppressor genes or hypermethylated in various cancer types, including urothelial carcinoma (Table 2). Nine of these genes had been shown to be associated with an aggressive phenotype in prior studies, some in multiple cancer types. Furthermore, some prior studies performed experimental validation of pathway alterations for many of these genes, including the estrogen signaling pathway.

**Table 2:** Hypermethylated genes by MethylMix criteria unique to patients who survived less than 2 years after cystectomy (references listed in Additional file 3: Table S3)

Gene symbol	Gene name	Chromosome	Associated with aggressive behavior in cancer	Tumor suppressor/hypermethylated in cancer	Altered pathways	Cancer types
CXCL6	C-X-C motif chemokine ligand 6	4q13.3		[44]		Ovarian cancer [44]
ZFP42	ZFP42 zinc finger protein	4q35.2			Sox2, NOTCH/STAT3 [45]	Human embryonic stem cell marker [45]
PITX1	Paired-like homeodomain 1	5q31.1	[46, 47]			Kidney cancer [46] Hepatocellular cancer [47]
RSPH9	Radial spoke head 9 homolog	6p21.1	[48]	[49]		*Bladder cancer* [50] Hepatocellular cancer
HIST1H3E	Histone cluster 1 H3 family member e	6p22.2	[51]			Glioma [51]
SP8	Sp8 transcription factor	7p21.2			Wnt/B-Catenin [52]	
TAC1	Tachykinin precursor 1	7q21-q22	[46, 53, 54]	[55]		Head/neck cancer [53] Colorectal cancer [54, 55] Esophageal cancer [46]
PON3	Paraoxonase 3	7q21.3		[56, 57]		Bladder cancer [56] Prostate cancer [57]
ABF1	Musculin (activated B cell factor 1)	8q13.3		[58]		Lymphoma [58]
FOXE1	Forkhead box E1	9q22	[59]	[60]	Estrogen signaling [61]	Colorectal carcinoma [59] Cutanous squamous cell cancer [60] Thyroid cancer [62]
CCDC67	Deuterosome assembly protein 1	11q21	[63]	[64]		Thyroid carcinoma [63] Gastric carcinoma [64]
ALX1	ALX homeobox 1	12q21.31	[65]	[66]	Snail [67]	Breast cancer [66] Non-small cell lung cancer [65] Ovarian cancer [67]
SLC6A15	Solute carrier family 6 member 15	12q21.31		[68, 69]		Colorectal carcinoma [68, 69]
EID3	EP300 interacting inhibitor of differentiation 3	12q23.3		[70]		Colorectal cancer [70]
NKX2-8	NK2 homeobox 8	14q13.3	[71]	[72, 73]	NF-KB [72], MEK/ERK [71]	*Bladder cancer* [71] Cervical cancer [73] Esopheageal cancer [72]
DIO3	Deiodinase, iodothyronine type III	14q32		[74, 75]		Lung cancer [74] Hematologic malignancies [75]
FOXC2	Forkhead box C2	16q24.1		[76]	MPK/AKT	Breast cancer [76]
HSPB6	Heat shock protein family B (small) member 6	19q13.13	[77]			Melanoma [77]
ZNF382	Zinc finger protein 382	19q13.13		[78]	NF-kB [79]	Esophageal cancer [78]

### Cumulative effects

**Fig. 3:**
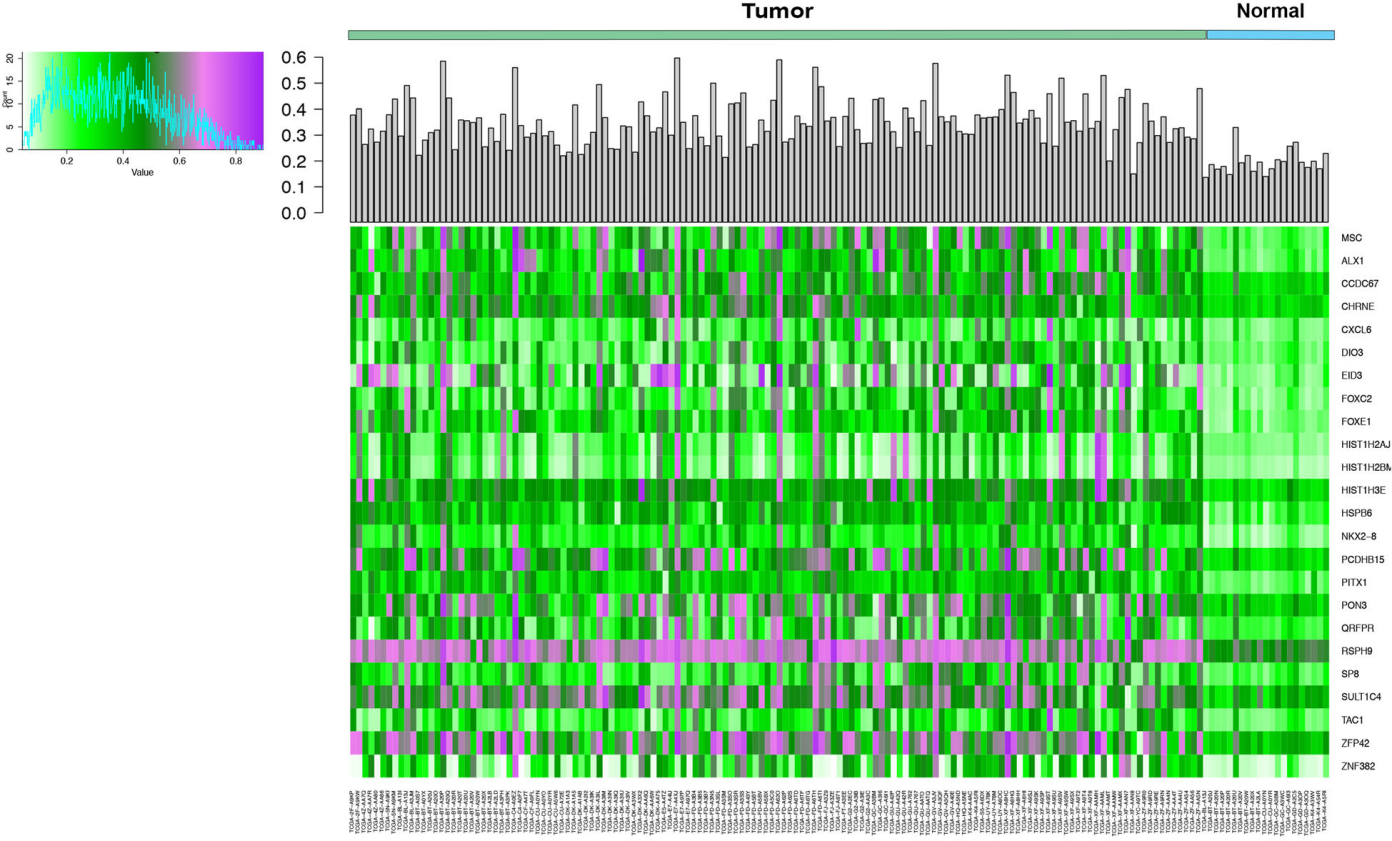
Heatmap of methylation values (beta values) for 24 genes uniquely hypermethylated in patients who survived less than 2 years after cystectomy. The gray bar histogram represents the mean beta value across all genes for each patient

While MethylMix describes each gene individually, we also wanted to evaluate the cumulated dose of hypermethylation across genes in any given patient. Thus, we assessed the effect of cumulative methylation changes for the 24 genes unique to patients with poor prognosis. Across the entire matrix of methylation values, the average methylation value (beta value) for normal samples for these genes was 0.23, whereas the average methylation value for tumor samples was 0.39 (*p* < 0.01). Figure 3 is a heatmap of the methylation values of all patients with survival of less than 2 years with poor prognosis genes selected. This heatmap demonstrates patients who survive less than 2 years may have hypermethylation of multiple genes associated with hypermethylation in various cancer types and/or poor prognosis.

### Pathway analysis

**Fig. 4:**
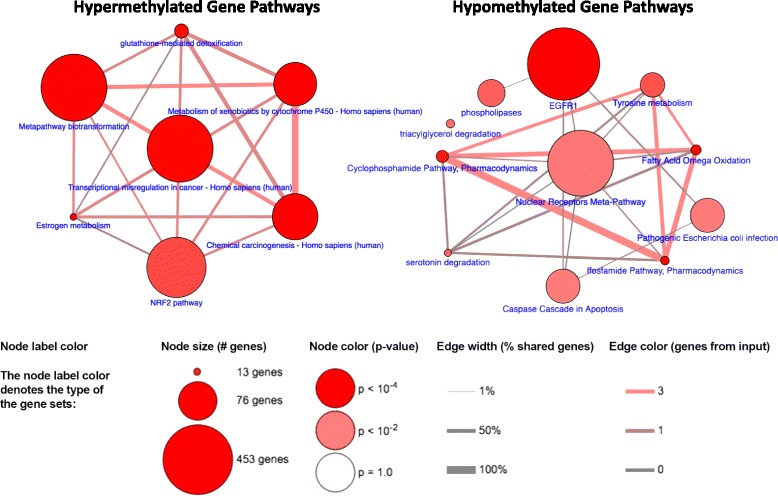
Select pathways enriched for genes hypermethylated and hypomethylated by MethylMix criteria in the analysis including all patients in the TCGA cohort. The full list of pathways is included in Additional file 2: Table S2

Figure 4 is a graphical representation of pathways enriched for genes significant by MethylMix criteria in the analysis that included all 408 patients. There were unique pathways represented by analysis of hypermethylated and hypomethylated genes. For hypermethylated genes, pathways involved in management of free radicals were found to be significant—both the glutathione and NRF2 pathways are key antioxidant pathways [6]. The estrogen metabolism pathway was also enriched in hypermethylated genes. The chemical carcinogenesis pathway was also hypermethylated, consistent with the relationship between bladder cancer and environmental exposures such as tobacco smoking and cyclic amines [22]. The most notable hypomethylated pathway was the EGFR1 pathway, a well-known oncogene that has been shown to be over-expressed in multiple cancer types [23]. Genes in the cyclophosphamide pathway were found to be hypomethylated—this agent can have significant effects on the urothelium causing hemorrhagic cystitis as well as delayed malignancy. There were also multiple pathways involved in fatty acid metabolism. Additional file 2: Table S2 lists detailed pathway analysis results by methylation status and patient group.

### Flow cytometry confirms the presence of EGFR

**Fig. 5:**
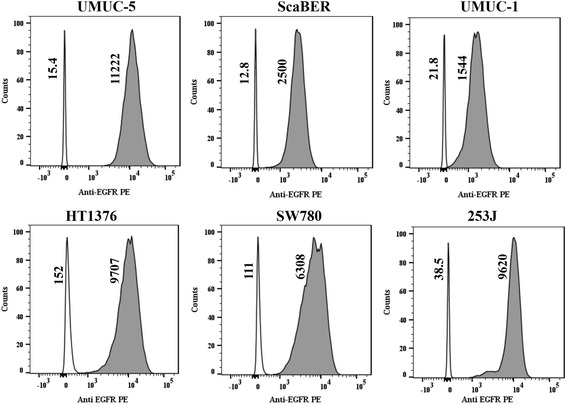
Flow cytometry analysis demonstrating expression of EGFR on the cell surface of various cell lines. The curve shaded gray represents binding of rat monoclonal antibody to human EGFR tagged with phycoerythrin (PE). The curve without shading (white) represents binding of an isotype control, which in the case was a PE-tagged monoclonal rat IgG2a kappa. The vertically written numbers next to each peak represent median fluorescence intensity for isotype control and anti-EGFR PE. For all cell lines tested (UMUC1, UMUC5, Scaber, HT1376, SW780, and 253 J), there was substantial EGFR expression compared with isotype control

There was a substantial amount of EGFR in all six cell lines tested, reflecting hypomethylation (Fig. 5). There was a mean increase of 83-fold over isotype control. The mean intensity for isotype control was 81.7 (range 12.5–154) while the EGFR mean intensity was 6816 (range 1544–11,222), *t* test *p* value < 0.01 (Fig. 5).

## Discussion

Epigenetic changes and modifications are an important part of carcinogenesis and subsequent tumor progression [24, 25]. Of the various epigenetic mechanisms, DNA methylation has been most studied and is classically associated with gene silencing via hypermethylation of CpG islands located in promoter regions of tumor suppressor genes. DNA hypomethylation has also been implicated in the development of cancer and likely results in genome rearrangement and chromosomal instability. Alterations in DNA methylation may be involved in the development of urothelial carcinoma of the bladder, with abnormalities identified in the normal urothelium of those who later develop frank cancer [5]. However, the methylation status of specific genes has been shown to be associated with worse prognosis [9, 10, 26], indicating that epigenetic changes may also be involved in tumor progression.

The earliest studies demonstrating the role of methylation in the development of urothelial carcinoma demonstrated a relationship between adverse clinical outcomes and the methylation state of promoters of specific genes known to be involved in the development of cancer [6, 7, 27]. These gene-specific studies demonstrated the relationship between increased methylation of specific gene promoters and grade [27], stage [28], and progression [7]. In addition to hypermethylation of specific genes, there was also evidence of an association between hypermethylation of multiple genes (characterized by a methylation index) and increased cancer aggressiveness [27].

The widespread use of high-throughput arrays created an opportunity to discover new genes involved in the epigenetic regulation of carcinoma [9, 29–31]. We sought to further elucidate the role and importance of methylation in bladder cancer by applying an integrative analysis tool to The Cancer Genome Atlas Project [32]. Although the TCGA has revealed the remarkable diversity of genetic alterations in bladder cancer, with only lung cancer harboring more mutations per megabase, it is clear that not all identified abnormalities contribute to the development of urothelial cancer, as many events deemed abnormal using high-throughput screening may have no biologic effect [33]. Fan et al. evaluated the relationship between methylation status in the promotor of 90 genes and RNA expression in six tissue types and found no correlation between methylation status and gene expression [34]. In bladder cancer, Lauss et al. demonstrated only 8% of methylated genes had an effect on gene expression [10]. When using high-throughput methodology with 450,000 probes, there is a need to distinguish the epigenetic alterations that act as effectors of the malignant phenotype from “passenger” alterations with no biologic effect. Thus, we used a model-based tool (MethylMix) to identify those genes with aberrant methylation and linked these data to RNA-seq data reflecting gene expression. This tool has been shown to produce results consistent with other methods of integrative analysis but also to produce unique findings [19]. The marriage of these complementary “omics” may aid in revealing biologically and clinically relevant information [11].

Our study corroborates findings from prior studies. We found increases in methylation of specific genes were associated with more aggressive disease [27, 35, 36], and we found a slightly greater proportion of genes being hypermethylated in the group who survived at least 2 years when compared to the group that died within 2 years of surgery. Although there was some overlap in statistically significant genes between patients with better versus worse survival, there were 24 genes that were unique to the group with worse survival. While the evaluation of these genes showed the majority were candidate tumor suppressor genes in a variety of cancers, this is the first time most of these genes have been associated with bladder cancer. This provides validation of the methodology and also provides an opportunity to evaluate the effect of cumulative methylation events. As a proof of principle, we show that many patients had hypermethylation of multiple poor-prognosis genes, suggesting that there may be many combinations of hypermethylation events that can lead to poor prognosis.

Our pathway analysis provided an opportunity to evaluate the gene lists produced by integrative analysis of all three patient groups, which is unique to our study. In examining all patients, one interesting finding from our pathway analysis was the enrichment of hypermethylated genes involved in antioxidant pathways. Genes involved in both glutathione-mediated detoxification as well as the nuclear factor (erythroid-derived)-like 2 (NRF2) pathway were statistically significantly hypermethylated, and both are integral in the cellular defense against oxidative stress. Glutathione is the most plentiful intracellular antioxidant and is a key component of redox-dependent regulation [37]. NRF2 is a transcription factor that serves as a key regulator in the cellular response to oxidative stress via induction of genes involved in the response to oxidative stress and xenobiotics, including those that regulate glutathione [38]. Deficiency of NRF2 has been shown to contribute to the development of cancer. In mice, knockout of the NRF2 gene increased susceptibility to formation of invasive bladder tumors in response to administration of a carcinogen [39].

In addition, there were two other notable findings. First, our pathway analysis enriched for hypermethylation of genes involved in the metabolism of estrogen. This is consistent with evidence for the role of estrogens in bladder cancer and suggests a basis for the observed clinical differences in the prevalence of bladder cancer between men and women. Shen et al. demonstrated that higher expression of estrogen receptor beta was associated with increasing stage and grade [40], and anti-estrogen compounds have been shown to inhibit the growth of bladder cancer cell lines [40, 41]. Targeting this pathway with anti-estrogens may have a therapeutic role in specific patients [42]. A second interesting observation was enrichment of hypomethylated genes associated with the epidermal growth factor receptor (EGFR) pathway. We were able to validate the presence of a substantial amount of EGFR on six bladder cancer cell lines. However, further validation of our findings is needed on independent cohorts.

Methylation is an attractive investigative tool for the study of aggressive cancer given that methylation is a reversible process. In myelodysplastic syndrome and AML, demethylating agents have been shown to have some effectiveness [43]. In this study, our integrative approach supports the findings of others showing that hypermethylation of specific genes is associated with aggressive urothelial carcinoma. The findings of this work may have application in the prevention of new disease and the reduction of disease recurrence in those with existing disease.

## Conclusions

Taken together, our integrative approach to identify biologically active methylation events has demonstrated anomalies in methylation in invasive urothelial carcinoma. Genes involved in oncologically relevant pathways including EGFR were found to be hypomethylated. We found a substantial increase in methylation in pathways involved in the management of free radicals including NRF2 and glutathione. It appears that multiple of genes with tumor suppressor activity may be associated with overall poor prognosis.

## Additional file

Additional file 1: Table S1. (XLSX 58 kb)

Additional file 2: Table S2. Full pathway list. (XLSX 55 kb)

Additional file 3: Table S3. (DOCX 20 kb)

Additional file 4: Table S4. (DOCX 13 kb)

## Abbreviations

TCGA: The Cancer Genome Atlas Project
