# Supplementary material for: Integrative analysis of the epigenetic basis of muscle-invasive urothelial carcinoma
Source: Clin Epigenetics. 2018 Feb 12;10:19. doi: 10.1186/s13148-018-0451-x (PMC5809922; doi:10.1186/s13148-018-0451-x)
Supplement: Supplementary file 3 — (DOCX 20 kb) [file 13148_2018_451_MOESM3_ESM.docx]

**Table S3**

**References For Table 2**

1. Menendez L, Walker D, Matyunina LV, Dickerson EB, Bowen NJ, Polavarapu N, et al. Identification of candidate methylation-responsive genes in ovarian cancer. Mol. Cancer. 2007;6:10.

2. Xu J, Sylvester R, Tighe AP, Chen S, Gudas LJ. Transcriptional activation of the suppressor of cytokine signaling-3 (SOCS-3) gene via STAT3 is increased in F9 REX1 (ZFP-42) knockout teratocarcinoma stem cells relative to wild-type cells. J. Mol. Biol. 2008;377:28–46.

3. Wei J-H, Haddad A, Wu K-J, Zhao H-W, Kapur P, Zhang Z-L, et al. A CpG-methylation-based assay to predict survival in clear cell renal cell carcinoma. Nat Commun. 2015;6:8699.

4. Calvisi DF, Ladu S, Conner EA, Seo D, Hsieh J-T, Factor VM, et al. Inactivation of Ras GTPase-activating proteins promotes unrestrained activity of wild-type Ras in human liver cancer. J. Hepatol. 2011;54:311–9.

5. Yoon H-Y, Kim Y-J, Kim JS, Kim Y-W, Kang HW, Kim WT, et al. RSPH9 methylation pattern as a prognostic indicator in patients with non-muscle invasive bladder cancer. Oncol. Rep. Spandidos Publications; 2016;35:1195–203.

6. Yamada N, Yasui K, Dohi O, Gen Y, Tomie A, Kitaichi T, et al. Genome-wide DNA methylation analysis in hepatocellular carcinoma. Oncol. Rep. Spandidos Publications; 2016;35:2228–36.

7. Yoon H-Y, Kim Y-J, Kim JS, Kim Y-W, Kang HW, Kim WT, et al. RSPH9 methylation pattern as a prognostic indicator in patients with non-muscle invasive bladder cancer. Oncol. Rep. 2016;35:1195–203.

8. Zhang Z, Tang H, Wang Z, Zhang B, Liu W, Lu H, et al. MiR-185 targets the DNA methyltransferases 1 and regulates global DNA methylation in human glioma. Mol. Cancer. BioMed Central; 2011;10:124.

9. Kennedy MW, Chalamalasetty RB, Thomas S, Garriock RJ, Jailwala P, Yamaguchi TP. Sp5 and Sp8 recruit β-catenin and Tcf1-Lef1 to select enhancers to activate Wnt target gene transcription. Proc. Natl. Acad. Sci. U.S.A. 2016;113:3545–50.

10. Misawa K, Mochizuki D, Imai A, Endo S, Mima M, Misawa Y, et al. Prognostic value of aberrant promoter hypermethylation of tumor-related genes in early-stage head and neck cancer. Oncotarget. Impact Journals; 2016;7:26087–98.

11. Tham C, Chew M, Soong R, Lim J, Ang M, Tang C, et al. Postoperative serum methylation levels of TAC1 and SEPT9 are independent predictors of recurrence and survival of patients with colorectal cancer. Cancer. 2014;120:3131–41.

12. Liu Y, Tham CK, Ong SYK, Ho KS, Lim JF, Chew MH, et al. Serum methylation levels of TAC1. SEPT9 and EYA4 as diagnostic markers for early colorectal cancers: a pilot study. Biomarkers. 2013;18:399–405.

13. Kitchen MO, Bryan RT, Emes RD, Glossop JR, Luscombe C, Cheng KK, et al. Quantitative genome-wide methylation analysis of high-grade non-muscle invasive bladder cancer. Epigenetics. 2016;11:237–46.

14. Shui IM, Wong C-J, Zhao S, Kolb S, Ebot EM, Geybels MS, et al. Prostate tumor DNA methylation is associated with cigarette smoking and adverse prostate cancer outcomes. Cancer. 2016;122:2168–77.

15. Ushmorov A, Leithäuser F, Ritz O, Barth TFE, Möller P, Wirth T. ABF-1 is frequently silenced by promoter methylation in follicular lymphoma, diffuse large B-cell lymphoma and Burkitt's lymphoma. Leukemia. 2008;22:1942–4.

16. Sugimachi K, Matsumura T, Shimamura T, Hirata H, Uchi R, Ueda M, et al. Aberrant Methylation of FOXE1 Contributes to a Poor Prognosis for Patients with Colorectal Cancer. Ann. Surg. Oncol. 2016.

17. Venza I, Visalli M, Tripodo B, De Grazia G, Loddo S, Teti D, et al. FOXE1 is a target for aberrant methylation in cutaneous squamous cell carcinoma. Br. J. Dermatol. 2010;162:1093–7.

18. Park E, Gong E-Y, Romanelli MG, Lee K. Suppression of estrogen receptor-alpha transactivation by thyroid transcription factor-2 in breast cancer cells. Biochem. Biophys. Res. Commun. 2012;421:532–7.

19. Landa I, Ruiz-Llorente S, Montero-Conde C, Inglada-Pérez L, Schiavi F, Leskelä S, et al. The variant rs1867277 in FOXE1 gene confers thyroid cancer susceptibility through the recruitment of USF1/USF2 transcription factors. PLoS Genet. 2009;5:e1000637.

20. Yin DT, Xu J, Lei M, Li H, Wang Y, Liu Z, et al. Characterization of the novel tumor-suppressor gene CCDC67 in papillary thyroid carcinoma. Oncotarget. Impact Journals; 2016;7:5830–41.

21. Park S-J, Jang H-R, Kim M, Kim J-H, Kwon O-H, Park J-L, et al. Epigenetic alteration of CCDC67 and its tumor suppressor function in gastric cancer. Carcinogenesis. 2012;33:1494–501.

22. Sandoval J, Mendez-Gonzalez J, Nadal E, Chen G, Carmona FJ, Sayols S, et al. A prognostic DNA methylation signature for stage I non-small-cell lung cancer. J. Clin. Oncol. 2013;31:4140–7.

23. de Groot JS, Pan X, Meeldijk J, van der Wall E, van Diest PJ, Moelans CB. Validation of DNA promoter hypermethylation biomarkers in breast cancer--a short report. Cell Oncol (Dordr). Springer Netherlands; 2014;37:297–303.

24. Yuan H, Kajiyama H, Ito S, Yoshikawa N, Hyodo T, Asano E, et al. ALX1 induces snail expression to promote epithelial-to-mesenchymal transition and invasion of ovarian cancer cells. Cancer Research. American Association for Cancer Research; 2013;73:1581–90.

25. Kim Y-H, Lee HC, Kim S-Y, Yeom YI, Ryu KJ, Min B-H, et al. Epigenomic analysis of aberrantly methylated genes in colorectal cancer identifies genes commonly affected by epigenetic alterations. Ann. Surg. Oncol. 2011;18:2338–47.

26. Mitchell SM, Ross JP, Drew HR, Ho T, Brown GS, Saunders NFW, et al. A panel of genes methylated with high frequency in colorectal cancer. BMC Cancer. BioMed Central; 2014;14:54.

27. Ashktorab H, Daremipouran M, Goel A, Varma S, Leavitt R, Sun X, et al. DNA methylome profiling identifies novel methylated genes in African American patients with colorectal neoplasia. Epigenetics. 2014;9:503–12.

28. Yu C, Zhang Z, Liao W, Zhao X, Liu L, Wu Y, et al. The tumor-suppressor gene Nkx2.8 suppresses bladder cancer proliferation through upregulation of FOXO3a and inhibition of the MEK/ERK signaling pathway. Carcinogenesis. Oxford University Press; 2012;33:678–86.

29. Lin C, Song L, Gong H, Liu A, Lin X, Wu J, et al. Nkx2-8 downregulation promotes angiogenesis and activates NF-κB in esophageal cancer. Cancer Research. American Association for Cancer Research; 2013;73:3638–48.

30. Steenbergen R, Ongenaert M. Methylation‐specific digital karyotyping of HPV16E6E7‐expressing human keratinocytes identifies novel methylation events in cervical carcinogenesis. The Journal of …. 2013.

31. Molina-Pinelo S, Salinas A, Moreno-Mata N, Ferrer I, Suarez R, Andrés-León E, et al. Impact of DLK1-DIO3 imprinted cluster hypomethylation in smoker patients with lung cancer. Oncotarget. 2016.

32. Martin-Subero JI, Ammerpohl O, Bibikova M, Wickham-Garcia E, Agirre X, Alvarez S, et al. A comprehensive microarray-based DNA methylation study of 367 hematological neoplasms. PLoS ONE. 2009;4:e6986.

33. Lindqvist BM, Wingren S, Motlagh PB, Nilsson TK. Whole genome DNA methylation signature of HER2-positive breast cancer. Epigenetics. 2014;9:1149–62.

34. Koga Y, Pelizzola M, Cheng E, Krauthammer M, Sznol M, Ariyan S, et al. Genome-wide screen of promoter methylation identifies novel markers in melanoma. Genome Res. 2009;19:1462–70.

35. Ma K, Cao B, Guo M. The detective, prognostic, and predictive value of DNA methylation in human esophageal squamous cell carcinoma. Clin Epigenetics. BioMed Central; 2016;8:43.

36. Cheng Y, Geng H, Cheng SH, Liang P, Bai Y, Li J, et al. KRAB zinc finger protein ZNF382 is a proapoptotic tumor suppressor that represses multiple oncogenes and is commonly silenced in multiple carcinomas. Cancer Research. 2010;70:6516–26.

**Hypermethylated Genes by MethylMix Criteria Unique To Patients who Survived Less than 2 years after Cystectomy: Novel Genes**

| Symbol | Full Gene Name | Chromosome |
| --- | --- | --- |
| SULT1C4 | sulfotransferase family 1C member 4 | 2q12.3 |
| QRFPR | pyroglutamylated RFamide peptide receptor | 4q27 |
| PCDHB15 | protocadherin beta 15 | 5q31 |
| HIST1H2AJ | histone cluster 1 H2A family member j | 6p22.1 |
| HIST1H2BM | histone cluster 1 H2B family member m | 6p22.1 |
| CHRNE | cholinergic receptor nicotinic epsilon subunit | 17p13.2 |
