# Supplementary material for: Integrative analysis of the epigenetic basis of muscle-invasive urothelial carcinoma
Source: Clin Epigenetics. 2018 Feb 12;10:19. doi: 10.1186/s13148-018-0451-x (PMC5809922; doi:10.1186/s13148-018-0451-x)
Supplement: Supplementary file 4 — (DOCX 13 kb) [file 13148_2018_451_MOESM4_ESM.docx]

Table S4:

| **Characteristic** | **Tumor**  **Mean(SD)** | **Normal =**  **Mean (SD)** | **p-value** |
| --- | --- | --- | --- |
| Cohort size | 408 | 20 |  |
| Mean age, years (SD) | 68.0 (10.6) | 69.9 (11.3) | 0.48 |
| Gender  Women (%)  Men (%) | 106 (26%)  302 (74%) | 9 (45%)  11 (55%) | 0.06 |
| T stage  pT2  pT3  PT4  NA | 120 (29%)  196 (48%)  59 (14%)  33 (8%) | 2 (10%)  5 (25%)  0 (0%)  13 (65%) | <0.01 |
